# Prevalence, Patterns and Correlates of Child and Adolescent Mental Disorders in Kumasi, Ghana: An Urban Community-Based Survey

**DOI:** 10.1007/s10578-024-01799-8

**Published:** 2024-12-14

**Authors:** Kwabena Kusi-Mensah, Kawther Inuwa, Caleb Otu-Ansah, Peggy Asiedu Ekremet, Ruth Charlotte Sackey, Ruth Owusu-Antwi, Sammy Ohene, Olayinka Omigbodun

**Affiliations:** 1 Department of Psychiatry, School of Clinical Medicine, University of Cambridge, Clifford Allbutt Building, Cambridge Biomedical Campus CB2 OAH, Cambridge, UK; 2 Department of Psychiatry, Komfo Anokye Teaching Hospital, Kumasi, Ghana; 3 School of Medicine and Dentistry, Department of Psychiatry, University of Ghana, Accra, Ghana; 4 University of New South Wales, Sidney, Australia; 5 Accra Psychiatric Hospital, Accra, Ghana; 6 College of Medicine, University of Ibadan, Ibadan, Nigeria

**Keywords:** Epidemiological survey, Child and adolescent mental health disorders, Community-based study, Prevalence data, Sub-Saharan Africa

## Abstract

This study examined the prevalence and correlates of mental disorders among youth in Kumasi, Ghana, through a community-based cross-sectional survey. 672 urban participants aged 6–17 years were surveyed. Mental disorders were screened using Rutter’s A2 Scale for Parent Assessment of Child Behaviour, with diagnoses confirmed by the Kiddie-Schedule for Affective Disorders and Schizophrenia. The Double Sampling method was used for weighted prevalence estimates, and correlates analysed using chi-square and logistic regression. Lifetime weighted prevalence of CAMH disorders was 30.4% (95% CI: 26.9–33.9), predominantly anxiety-related disorders, with current weighted prevalence 18.6% (95% CI: 15.7–21.5). Notably, lacking an active reading habit was associated with nearly three times the odds of mental illness. Children in the 3rd and 4th wealth quintiles had significantly higher odds of mental disorder (12- and 9-times increased odds, respectively), as did lack of caregiver homework supervision among children under 11 years. This study provides the first community-based prevalence figures for childhood mental disorders in Ghana, highlighting the link between poverty-related factors and mental health, and suggesting potential policy interventions to inform policy.

## Introduction

Increasingly, mental health has taken a place of global importance in health-planning around the world, particularly because of its impact on child growth and development [1–3] and the changing age demographics of the world, with an increasing youthful bulge in low-and-middle-income countries (LMICs) as the more affluent high-income countries (HICs) sees population decline. Sustainable Development Goal (SDG) 3 which targets health and well-being, places emphasis an on addressing the growing burden of mental health [2, 4]. Up to 50% of adult mental disorders begin before the age of 14 years and 75% before the age of 24 years [5–7]. These cause immense distress loss of productivity with neuropsychiatric disorders in young people accounting for up to 45% of disability-adjusted life years (DALYs) lost [8]. Meanwhile, many LMICs, such as Ghana, have youthful populations, with children increasingly surviving beyond age five in these countries due to improving economies and infant mortality figures, which has resulted in a growing shift toward non-communicable diseases such as child and adolescent mental health (CAMH) disorders [9], indicating the importance of focusing on child and adolescent mental health.

In the mid-2000s, the World Health Organization (WHO) initiated the 'Child and Adolescent Mental Health Resources Atlas Project' to assess global CAMH resources [10]. This project estimated the prevalence of childhood psychopathology to be between 10 and 20% globally [10]. Several well-designed studies supported this prevalence range: an older systematic review of papers published from 1995 to 2005 reported a mean worldwide prevalence of CAMH disorders at 12% [11], while a more recent systematic review from 1985 to 2012 found a mean worldwide prevalence of 13.4% [12]. In the latter review, anxiety-related disorders had the highest global prevalence at 6.5%, followed by 'disruptive disorders' at 5.7%, with attention-deficit hyperactivity disorder (ADHD) accounting for 3.4%, and depressive disorders at 2.6% [12]. A meta-analysis confirmed similar prevalence rates of CAMH disorders in LMICs as in high-income countries (HICs) based on the limited studies available [13, 14]. There have been reported some differences in urban and rural prevalence data such as was found in China [15] Unfortunately, relatively little data on Child and Adolescent Mental Health (CAMH) Disorders exists for sub-Saharan Africa in general, and Ghana in particular [13, 14, 16].

Further, there is a dearth of community-based prevalence studies on CAMH disorders emanating from low- and middle-income countries (LMIC), especially in sub-Saharan Africa and in particular among children [12–14]. In sub-Saharan Africa, much of the data published on child psychopathology were school based studies, primary health care or hospital-based data [17–21] with not many community-based surveys found. In Ghana, to the best of authors’ knowledge, no literature on community-based CAMH survey could be found, with only a school-based survey found which placed current prevalence of CAMH disorders (CAMHD) at 7.1% [22]. This can be a problem as not all children are necessarily in school in Africa, and not all CAMH problems may necessarily report to hospital for various reasons, affecting the accuracy of such non-community-based studies.

Several sociodemographic factors have been reported as affecting definition of CAMHD acronym has now been introduced above in the immediate preceding sentence. Factors such as parental education [13, 14, 23–25], family structure [23–30], marital status and stability [24, 25, 27] exhibit associations with childhood psychopathology. Other ecological factors that also affect children’s mental health include school-related factors such as repetition of a schoolyear [28], bullying [31, 32], and parental educational attainment [13, 14]. Again, these may differ by urban and rural centres, with children in rural China for example being more vulnerable to various risk factors compared to their urban compatriots [15], while others report an increase in risk factors such as urban food insecurity, lacking access to safe water and violence among urban adolescents [33, 34].

The issue however is that, sociodemographic correlates affecting CAMHD vary greatly in different parts of the world. For example, while in HICs boys had significantly higher CAMH disorders than girls before puberty [24, 25, 35]], in sub-Saharan Africa no such significant difference was found in a meta-analysis [27]. Other such differences in sociodemographic correlates have been reported in US-based studies [26], studies from Italy [23] and the United Kingdom [24, 25], underscoring the need for an exploration of local sociodemographic correlates of CAMHD in Ghana.

The United Nations Development Programme (UNDP) has advanced a concept termed “accelerators”, which refers to practical actions or services that concurrently influence multiple SDGs [36–38] and which when combined have synergistic effect across several SDGs [36]. Some suggested accelerators include promoting active reading for children, cash transfers for parents, and adequate supervision of children [39, 40] which appear to enhance various SDGs including mental health. For LMICs, where mental health care is allotted 1% of national budgets [2], it is vital that research explores these social and economic factors which act as risk or resilience factors for CAMH that may be targeted for intervention. This may be more cost-effective, resourceful, and fruitful in meeting other SDG goals. This therefore is another strong impetus for uncovering the local predictive factors for CAMH disorders in Ghana, which could also be funnelled into designing interventions based on the concept of “accelerators” that could have an exponential effect on attaining the SDGs such as SDG 3 on optimum health.

Meanwhile, the majority of CAMH research is conducted in HICs [41–44] with only 10% of research coming from LMICs [11], even though over 80–90% of the world's children reside here [45, 46]. There is thus an urgent need to deal with the dearth of research on CAMH in sub-Saharan Africa and Ghana specifically as this impedes CAMH policy development and the implementation and establishment of services [16] and population-level interventions for a large proportion of the world’s children residing in this most youthful region of the world. This study could contribute to a useful starting point for addressing childhood mental challenges in this region of the world.

This study sought to bridge the data gap on the dearth of local information on CAMH disorders by providing community-based prevalence data on CAMH disorders among the general population and give mental health professionals who work with children and adolescents in Ghana (and by extension sub-Saharan Africa) a better understanding of who is at risk for various mental disorders and what factors should be encouraged to help clients and their families. The study aims to determine the prevalence of mental disorders in Ghanaian children and their sociodemographic and ecological (household) correlates.

## Methods

### Study Design

This study was a community-based cross-sectional epidemiological prevalence study. It was part of a larger comparative cross-sectional study comparing ‘left-behind children’ (LBCs- children left behind with other caregivers after one or both parents have migrated) with ‘non-left behind children’ (NLBCs) in terms of the variables under study in the current paper. This paper however reports the results of the first part of this study: epidemiological survey of the entire general population (including left-behind and non-left behind children), before further analysis was done on the sub-populations of LBCs vs NLBC controls.

### Study Location

This study was conducted in Ghana's Ashanti region, the most populous and urbanized region outside the capital [47]. The Ashanti region was chosen as it is among Ghana's most multi-ethnic and populous areas [48, 49]. The specific site was the city of Kumasi, the second largest city in Ghana which makes up over 40% of the Ashanti region’s population [48, 49]. Within Kumasi, the Fante New Town community was purposively selected. This is a typical dense inner-city area populated largely but not exclusively by urban poor with sizeable minority of middle-class housing. It was chosen as representative of Kumasi's urban core [48] so findings could be generalized. The Fante New Town community specifically offered a glimpse of life for Ghana's urban poor in one of its fastest growing cities.

### Participants

Any child aged 6–17 years who gave documented assent along with a guardian/caregiver who gave written informed consent was included in the study. Children with an intellectual or physical disability that prevented them from being able to answer questions themselves and provide information were excluded. The calculated sample size was 632 although a total of 730 participants were recruited into the study. Of these, 12 participants withdrew consent during the assessment process and 46 participants failed to complete all assessment questionnaires and instruments giving a total of 58 out of 730 recruited participants who dropped out of the study. This gave a response rate of 92.1%, with 672 participants completing all aspects of the assessment.

### Sampling Technique

Potential households were selected by systematic random sampling method. A household was defined as a person or group of persons who lived together in the same house or compound and shared the same income source(s) and housekeeping arrangements. Records from the local government office indicated that Fante New Town had a population of 28,100 spread across 8,162 households occupying 1,913 housing units/compounds [48]. Sharing the rounded off sample size of 640 equally among the 8162 households, simple direct proportion translated into a target of 186 households occupying approximately 44 housing units to be visited. A list of house numbers was obtained from the Kumasi Metropolitan Assembly and the Subin Sub-metro office, and the index house randomly selected by a computer-generated random number. After that, every fortieth house was selected for participation to give each house/household in the community an equal chance of being selected. If a selected housing unit had no household with an eligible child or adolescent, the next house was approached. If a selected household had only one child or one adolescent, the child or adolescent was recruited automatically. If consent or assent was refused, the next eligible household was moved to, to replace the refusing household. In every selected household which had more than one child or adolescent, only one was randomly selected by balloting.

### Measures

#### Socio-Demographic Questionnaire

A modified version of the Socio-demographic Questionnaire [19, 20] consisting of questions relating to socio-demographic and ecological characteristics was utilized. It was also modified to incorporate the International Wealth Index instrument to assess the standard of living.

#### The International Wealth Index

(IWI) [50] is a measure of long-term household economic status using a simple twelve-item set of durable assets possession (such as television and bicycles), access to basic services (. e.g., electricity) and housing material. This provided an accurate and validated estimation of material well-being and socioeconomic status. The Wealth Index is based on data from 2.1 million households in 97 LMICS countries (including 3 surveys from Ghana).

#### Rutter’s A2 Scale for Parent Assessment of Child Behaviour

Overall mental health was assessed using Rutter’s Parent Questionnaire developed by Rutter [51]. This is a 31-item behavioural screening questionnaire to identify children who are at high risk for a mental disorder. It was validated in Nigeria in a similar environment to the study site [52, 53], and has been used extensively in many parts of the world [54, 55]. Test–retest, inter-rater reliability and stability tests on this scale have yielded scores of 0.74, 0.64 and 0.73, respectively [51]. A threshold of 7 had been recommended by Omigbodun [53] as the best trade-off between high sensitivity (0.61) and a low false-positive rate (specificity: 0.74) for this environment, thus this cut-off was adopted for this study. Children scoring in the abnormal range (≥ 7), and 10% of those scoring below this range, were assessed using the K-SADS-PL DSM 5 (see next line).

#### Kiddie- Schedule for Affective Disorders and Schizophrenia version 2016 (K-SADS-PL DSM 5)

The K-SADS-PL DSM 5 (Kaufman et al., 1997) is a semi-structured interviewer-administered diagnostic interview instrument designed to assess lifetime (current and past) episodes of psychiatric disorders in children and adolescents aged 6–18 years according to DSM-5 criteria. It is administered to children, parents, or teachers to generate summary ratings. This instrument has two parts; the diagnostic screening part which surveys for and rates the primary symptoms of disorders and the diagnostic supplement part in which children who score above threshold during screening are assessed for the diagnosis of current and most severe past psychiatric episodes. In this study, the diagnostic supplement part was used by the primary investigator (PI) to make a diagnosis of present or severe past episode of DSM-5 disorder in children screened on Rutter’s A2 Scale screener (see Data Collection for further details on how K-SADS was used).

### Adaptation and Administration of Study Instruments

All instruments were translated into the local dialect, Twi, using the forward- back-translation method [56]. First, a mental health professional, fluent in both Twi and English, translated the instrument into Twi. Then a physician with knowledge of mental disorders, with fluency in both Twi and English, who was unfamiliar with the instruments, translated them back to English. The two English translations were then compared item by item for a high degree of similarity, with the closest translation to the original item selected. For the K-SADS-PL, because this was used by a single trained psychiatrist (the lead author) who himself is natively fluent in the local language and culturally competent in the local setting of the study, communication of the concepts underlying each item/question on the instrument (i.e. ensuring conceptual equivalence) was relatively straightforward to do.

### Study Procedure

Contact was made with local government officials to seek their cooperation and expert knowledge of the area. Six research assistants (RAs) who spoke English and Twi fluently were recruited and trained in the theoretical basis and practical administration of the various instruments. Ethical approval was sought and obtained from the Committee for Human Research, Publications and Ethics (CHRPE) of the Kwame Nkrumah University of Science and Technology (KNUST). Informed consent and assent were sought and documented for all participants selected for the study. The content of the consent forms was translated into Twi for guardians who did not speak English in the presence of a witness who had read, understood and signed the content. Most interviews were conducted in the local dialect Twi, which all the RA’s and the assessing psychiatrist were natively fluent in. All study participants were given a token gift of a pen, pencil, and jotter, while caregivers were given token mobile recharge cards as appreciation for their time and participation. Issues of inherent power-differential problems were mitigated by the study team being intentional about making every effort to get participants to relax and disabuse their minds of any notion of “superiority vs inferiority” or “right or wrong answers” prior to commencing the interview and respecting cultural norms.

### Data Collection

**Fig. 1:**
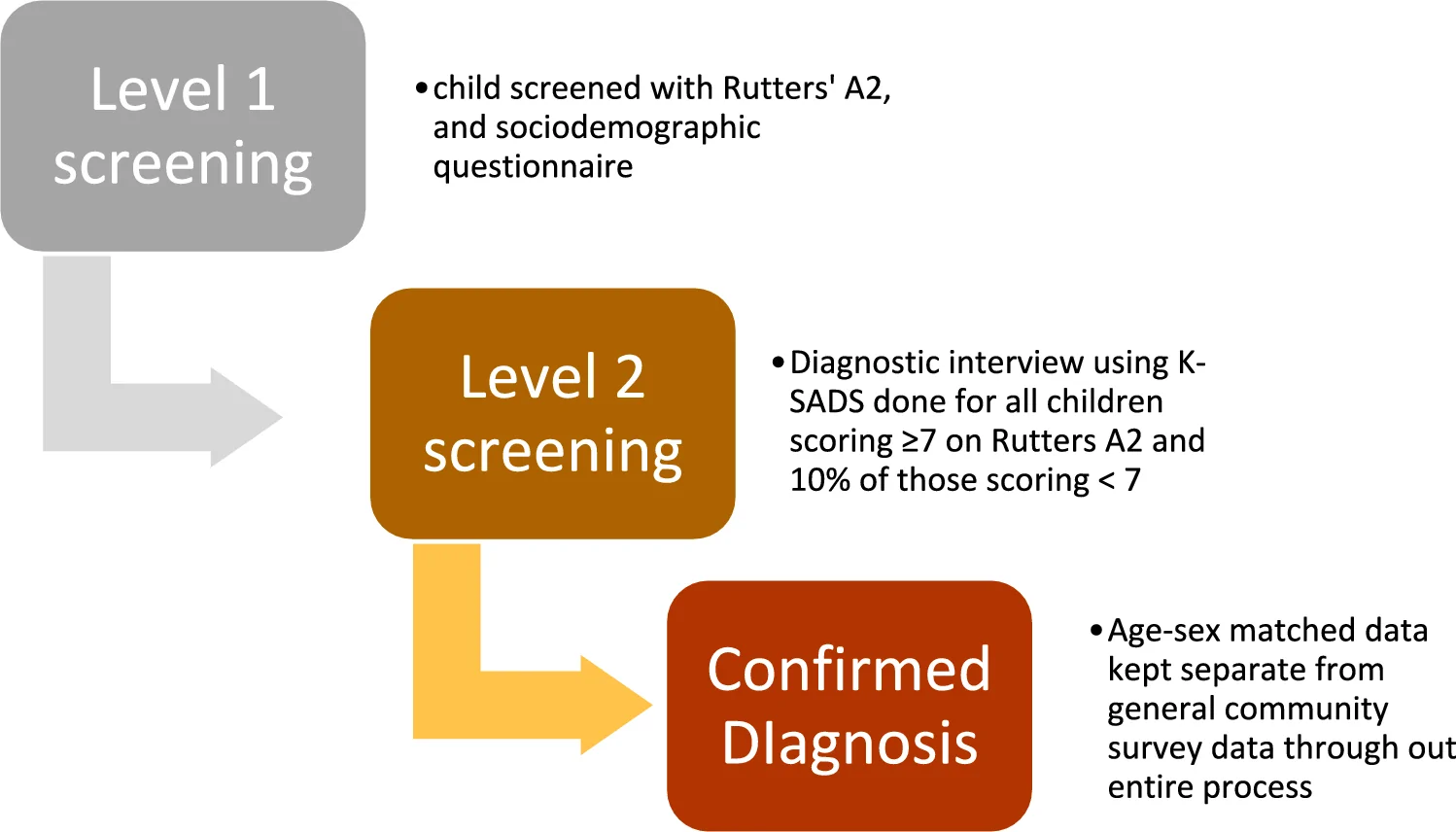
Data collection Process flowchart

A one-day pre-test study was conducted on a small sample (N = 15) of children and adolescents within the study sample age bracket in the Bantama community, an inner-city urban-poor community with similar demographics and ecological characteristics as the study site. The data collection process is summarised in a flowchart in Fig. 1 below.

The interviews were conducted after school and work hours on weekdays, between four pm and eight pm, and at weekends to increase the chance that children and their caregivers would be found at home. The RAs were organised into three two-person pairs, with each pair completing an average of 6 surveys per day. The PI played a daily supervisory role and administered the K-SADS-PL to participants who scored at or above the cut-off score of seven on the Rutter’s A2 scale. In the first phase of the interview procedure, once a household had been approached and it had been determined that a child aged 6–17 years lived there, informed consent and assent was obtained and the screening commenced. The PI promptly visited any household where a child scored at or above the cut-off of seven for a full diagnostic interview with both the caregiver and the child. The diagnostic interviews took between 40–60 min on average to conduct. Where a diagnosis was confirmed, psychoeducation was done for the child and caregiver, and a referral note was given to the caregiver to access formal healthcare at the Department of Psychiatry of the Komfo Anokye Teaching Hospital, along with the contact number of the PI for any subsequent clarifications or assistance.

The systematic random sampling continued (as described above) until the minimum sample size was surveyed and exceeded. Further, 10% of children who screened negative (i.e., below the cut-off of seven) were randomly selected and approached to partake in a full diagnostic interview using the K-SADS-PL as above. For every participant who scored seven or above, the next participant who scored below seven was interviewed. This aimed to capture false negatives screening below the cut-off who indeed had a mental disorder, providing a more accurate community prevalence.

### Statistical Analysis

The data were analysed using R [57, 58]. The prevalence of mental disorders identified with the Kiddie-SADS was calculated for the total sample using the Double Sampling method [59] as used in similar settings [18, 53]. The score distribution on the Rutter Scale A2 for all children with a mental disorder based on the Kiddie-SADS interview was determined.

In each score band, the proportion of individuals with a mental disorder at the second stage was then multiplied by the number of scorers in this score band at the first stage. By summing up these values for every score band, the estimated number of children with mental disorders was obtained. The value was then expressed as a percentage of the total population of children screened to obtain the total estimated weighted prevalence of mental disorders in the study sample. This method of weighting was also applied to each diagnostic category as follows: the occurrence of individual mental disorders was simply counted, estimated (as just described) and derived as a percentage of the total sample population, regardless of overlap with other co-morbidities within the same individual child. So here, the unit of calculation used was the mental disorder over the total population (as opposed to the number of individuals over the population).

For the bivariate analysis for association of sociodemographic and ecological variables with the presence of mental disorder, Chi-square analysis at a significance value of 5% was done. Where the values in the individual cells were so small that expected value was ≤ 5 in any cell, Fisher’s exact test was chosen to give more accurate results [60], using the Monte Carlo method to obtain a good approximation [61]. We then constructed a multivariate logistic regression model using both theory and variables that showed statistical significance in the bivariate analysis, while also utilising Odds Ratio at a significance level of 5% and 95% confidence interval to estimate effect size. To select the best logistic model for the given data set (for most accurate results), the ‘residual deviance’ and Akaike Information Criterion (AIC) number [62] for each logistic model were compared after excluding each of the above variables in turn, to determine which of them contributed the least significantly to the accuracy of the model and should therefore be appropriately removed from the model.

## Results

### Descriptive Characteristics of the Study Sample

Table 1 below summarises the personal characteristics of the study participants. There were 344 females (51.2%) with a mean age of 11.6 (*SD* = 3.14) years, media age of 12 years and modal age of 13 years. With regards to religious affiliation and commitment, 638 (94.9%) reported that the teachings of their religion ‘much’ or ‘very much’ guided their behaviour. 18 (2.7%) reported engaging in an income-generating activity of which 15 (83.3%) were street-hawkers (see supplementary material for definitions of the sociodemographic and ecological variables/terms). Table 1 also shows the ecological (parent and household) characteristics of the study population. A quarter (25.1%) of fathers had tertiary education and 30 (4.5%) had no formal education while 81 (12.1%) mothers had tertiary education and 68 (10.1%) had no formal education. Five hundred and sixty-three (83.8%) reported that both biological parents were married to each other. Two hundred and sixty (38.7%) respondents had a standard of living (as measured by the IWI score) in the highest quintile (IWI score above 80 and above) (see supplementary material for wealth quintile definitions). Three hundred and eighty-seven (57.6%) reported an active reading habit in their leisure time, and 346 (51.5%) reported having access to the Internet. Finally, 33 (4.9%) participants reported they had repeated a class. 324 (48.3%) participants reported frequent and regular homework supervision by caregivers (i.e., “always”, “mostly” or “sometimes”) and 347 participants (51.7%) reported infrequent supervision (“hardly” or “never”). Further analysis revealed that of those in the ‘infrequent supervision’ category, 14 out of 347 (4.0%) were below the age of 11 years (primary school), while 333 (96%) were 11 years old and above (secondary school) (not shown in table).

**Table 1:** Personal socio demographic, ecological and school characteristics of study population (N = 672)

Variable	Freq. (%)	Variable	Freq. (%)	Variable	Freq. (%)	Variable	Freq. (%)
Sex		Practice of religion		Family history of mental illness		Paid work	
Male	328 (48.8)	Yes	663 (98.7)	Yes	26 (3.9)	Yes	18 (2.7)
Female	344 (51.2)	No	9 (1.3)	No	646 (96.1)	No	654 (97.3)

Age in categories		Type of religion		Relative* with mental illness n = 26		What type of work n = 18	
Pre-adol. (6–9 years)	192 (28.6)						
Early-adol. (10–12 yrs)	199 (29.6)	Islam	275 (40.9)	1st degree rel	2 (7.7)	Hawking	15 (83.3)
Mid-adol. (13- 15 yrs)	198 (29.5)	Christianity	388 (57.7)	2nd degree rel	20 (76.9)	Artisans^*^	2 (11.1)
Late-adol. (16 – 17 yrs)	83 (12.4)	No religion	9 (1.3)	3rd degree rel	4 (15.4)	Other^*^	1 (5.6)

Father’s level of education		Mother’s education		Parental marital status		Wealth index score categories	
No formal education	30 (4.5)	None	68 (10.1)				
Basic	175 (26.0)	Basic	297 (44.2)	Married	563 (83.8)	80.1–100	260 (38.7)
Secondary	276 (41.1)	Secondary	219 (32.6)	Separated/divorced	67 (10.0)	60.1–80	312 (46.4)
Tertiary	169 (25.1)	Tertiary	81 (12.1)	Never married	17 (2.5)	40.1–60	95 (14.1)
Unknown	22 (3.3)	Unknown	7 (1.0)	Widowed	25 (3.7)	≤ 40	5 (0.7)

Active reading habit		Internet access		School type		Repeated a class	
Yes	387 (57.6)	Yes	346 (51.5)	Private	298 (44.4)	Yes	33 (4.9)
No	285 (42.4)	No	326 (48.5)	Public	373 (55.6)	No	638 (95.1)

School area of location		Language of instruction		No. times repeated n = 33		Homework supervision n = 671^&^	
				Once	27 (81.8)		
Urban affluent neighbourhood	75 (11.1)	English	613 (91.4)	Twice	5 (15.1)	Frequent superv^§^	324 (48.3)
Urban poor neighbourhood	596 (89.0)	Twi	58 (8.6)	Three times	1 (3.0)	Infrequent superv^§^	347 (51.7)

*Relatives: 1st Degree relative = parents, sibling; 2nd degree relatives- uncle/aunt, grand-parents; 3rd degree relatives = cousins, others; ^+^Artisans include = Carpentry, mechanic, Other = shop attendant; ^§^Frequent supervision = ‘always’, ‘mostly’, sometimes’; ^§^Infrequent supervision = Rarely, never; ^&^ *Note.* ^&^the change from 672 to 671 is because one child reported not attending school

### Weighted Prevalence of mental disorders among general population

Table two shows the number of participants in each score band on the Rutter’s A2; the number at each score-band who were assessed at the second stage K-SADS diagnostic interview; the number of K-SADS “cases” (i.e. those confirmed with a definitive diagnosis of a mental disorder); and the estimated number of participants with a mental disorder in the entire sample population for each score band as calculated using the weighted proportion of each score band. 47 participants scored at or above the cut-off of 7 and 39 gave consent for and completed the K-SADS-PL assessment. Fifty-two participants who screened below the cut-off of 7 consented for a level 2 diagnostic interview, making 91 participants who completed interview with the K-SADS-PL. Thirty-seven participants of 91 assessed at 2nd stage had a mental disorder. Thus 204 (30.4%) of 672 were estimated to have had a mental disorder (Table 2).

**Table 2:** Weighted Estimate of Participants with Mental Disorders per Rutter’s A2 score-band Following Screening (N = 672)

Level 1 screening with Rutter’s A2		Level 2 screening with K-SADS-PL		
Rutter’s score	Number scoring this rutter’s score	Number assessed with K-SADS	Confirmed “cases” (weighting as %)	Estimated no. with a CAMHD per score band
0	36	2	0 (0)	0
1	71	5	0 (0)	0
2	126	11	5 (45.5)	57
3	153	13	4 (30.8)	47
4	110	10	3 (30.0)	33
5	78	6	1 (16.7)	13
6	51	5	3 (60.0)	30
7	6	3	1 (33.3)	2
8	9	8	3 (37.5)	3
9	9	7	2 (28.6)	2
10	2	2	1 (50.0)	1
11	3	3	0 (0)	0
12	1	1	1 (100)	1
13	2	2	1 (50.0)	1
14	4	4	3 (75.0)	3
15	1	1	1 (100)	1
16	2	1	1 (100)	2
17	2	2	2 (100)	2
21	3	2	2 (100)	3
23	2	2	2 (100)	2
33	1	1	1 (100)	0
Total	672	91	37	**204**

Table 3 shows the estimated life-time weighted prevalence of various mental disorders. ‘Single disorder’ refers to cases where the participant in question was diagnosed with one single diagnosis, while ‘mixed disorders’ refers to a diagnosis with 2 or more disorders in a single child. Here, the unit of calculation used was the person/child with the disorder (i.e., number of children with disorders over total sample). In total, 37 participants had a confirmed mental disorder, with an estimated 204 expected to have a mental disorder. This gave an estimated life-time weighted prevalence of 30.4% (95% CI: 26.9–33.9).

**Table 3:** Lifetime weighted prevalence of specific DSM 5 (K-SADS confirmed) disorder (N = 672)

Single disorder	Actual number	Estimated number	Estimated prevalence % (95% ci)
ADHD (combined type)	4	14	2.08 (1.0–3.2)
Panic disorder	3	23	3.42 (2.0–4.8)
Specific phobia (darkness)	1	1	0.15 (− 0.1–0.4)
OCD (combined type)	1	11	1.64 (0.7–2.6)
Separation anxiety dis	2	12	1.79 (0.8–2.8)
PTSD	1	11	1.64 (0.7–2.6)
Enuresis	2	12	1.79 (0.8–2.8)
Oppositional defiant disorder	2	3	0.45 (− 0.1–1.0)
Tic disorder	1	10	1.49 (0.6–2.4)
Total number with single disorder	17	97	14.5 (11.8–17.2)

Mixed disorders	Actual number	Estimated number	Estimated prevalence % (95% ci)
ADHD & ODD	1	1	0.15 (− 0.1–0.4)
ADHD & ODD & Enuresis	2	2	0.3 (− 0.1–0.7)
Agoraphobia & panic disorder	1	11	1.64 (0.7–2.6)
Anorexia Nervosa &Moderate Depressive Disorder	1	11	1.64 (0.7–2.6)
Bipolar I & Panic Dis. & PTSD	1	1	0.15 (− 0.1–0.4)
Conduct Disorder & Polysubstance Use Disorder	1	1	0.15 (− 0.1–0.4)
Major Depressive Disorder & Dysthymia	1	1	0.15 (− 0.1–0.4)
Major Depressive Dis. & Social Anxiety Dis. & Separation Anxiety Dis. & GAD	1	12	1.79 (0.8–2.8)
Sleep Terrors & PTSD & Sexual Abuse	1	1	0.15 (− 0.1–0.4)
PTSD & Major Depressive Dis. & Sexual Abuse	1	11	1.64 (0.7–2.6)
Panic Dis. & Separation Anxiety Dis	1	13	1.93 (0.9–3.0)
Panic Dis. & Separation Anxiety Dis. & Bipolar I Dis	1	12	1.79 (0.8–2.8)
Enuresis & Dysthymia	1	1	0.15 (− 0.1–0.4)
Enuresis & Encopresis & OCD & ODD	1	1	0.15 (− 0.1–0.4)
Enuresis & ODD	2	3	0.45 (− 0.1–1.0)
Separation Anxiety Dis. & Specific Phobia	1	12	1.79 (0.8–2.8)
Social Anxiety Dis. & GAD	1	12	1.79 (0.8–2.8)
Social Anxiety & Specific Phobia & Binge-Eating Dis	1	1	0.15 (− 0.1–1.0)
Total number with mixed disorder	20	107	15.9 (13.1–18.7)
Total number with disorder	37	204	30.41 (26.9–33.9)
Total number without disorder	54	468	69.64 (66.2–73.1)
Total	91	672	100

Table 4 shows the estimated current prevalence within the past 12 months prior to interview of specific child and adolescent mental disorders, broken down by single disorders and mixed disorders. The total number with current mental illness was 29, with 125 estimated to have mental disorders, making an estimated weighted current prevalence of 18.6% (95% CI: 15.7–21.5).

**Table 4:** Current weighted prevalence of specific DSM-5 (K-SADS confirmed) disorder (N = 672)

Single disorder	Actual no	Est. no	Est. prevalence %
ADHD	5	15	2.23 (1.1–3.3)
Panic Disorder	2	13	1.93 (0.9–3.0)
OCD (Combined Type)	1	11	1.64 (0.7–2.63)
Separation Anxiety Dis	1	1	0.15 (− 0.1–0.4)
Dysthymia	1	1	0.15 (− 0.1–0.4)
Enuresis	2	12	1.79 (0.8–2.8)
Sleep Terrors	1	1	0.15 (− 0.1–0.4)
Oppositional Defiant Disorder	2	3	0.45 (− 0.1–1.0)
Tic Disorder	1	10	1.49 (0.6–2.4)
Total number	16	67	9.98 (7.7–12.2)

Mixed disorders	Actual no	Est. no	Est. prevalence %
ADHD & ODD	2	2	0.30 (− 0.1–0.7)
Agoraphobia &Panic Dis	1	11	1.64 (0.7–2.6)
Bipolar I & Panic Dis. & PTSD	1	1	0.15 (− 0.1–0.4)
Conduct Dis. & Polysubstance Use Dis	1	1	0.15 (− 0.1–0.4)
Major Depressive Dis. & Social Anxiety Dis. & Separation Anxiety Dis. & GAD	1	12	1.79 (0.8–2.8)
Panic Dis. & Separation Anxiety Dis	1	13	1.93 (0.9–3.0)
Panic Dis. & Separation Anxiety Dis. & Bipolar I Dis	1	12	1.79 (0.8–2.8)
Enuresis & Dysthymia	1	1	0.15 (− 0.1–0.4)
Enuresis & Encopresis & OCD & ODD	1	1	0.15 (− 0.1–0.4)
Enuresis & ODD	2	3	0.45 (− 0.1–1.0)
Social Anxiety & Specific Phobia & Binge-Eating Dis	1	1	0.15 (− 0.1–0.4)
Total number with mixed disorder	13	58	8.63 (6.5–10.8)
Total no. with current disorder	29	125	18.61 (15.7–21.6)
Total no. without current disorder	62	547	81.40 (78.5–84.3)
Total	91	672	100

In Table 5 below, individual disorders and their estimated weighted lifetime prevalence are shown (i.e., estimated number of disorders over total sample). There were 7 cases of ADHD (combining both single and mixed diagnoses), with a weighted estimate of 18 ADHDs in the entire sample, giving an estimated weighted lifetime prevalence of 2.7% (95% CI: 1.5–3.9) of the sample having ADHD. With regards to anxiety-related disorders, 31 participants had a K-SADS confirmed anxiety disorder, and an estimated weighted lifetime prevalence of 244 (36.4%: 95% CI: 32.7–39.9) participants. There were 7 individuals with enuresis, giving an estimated weighted lifetime prevalence of 18 (2.7%: 95% CI: 1.5–3.9) participants.

**Table 5:** Lifetime prevalence of individual disorders (including those with overlapping diagnoses) in study sample (N = 672)

Disorder	Actual No	Est. No	Est. Prevalence %
Generalized Anxiety Disorder Panic Disorder Specific Phobias (2 Darkness, 1 Animal Type) Agoraphobia Obsessive Compulsive Dis. (1 Obsessive, 1 Combined) Separation Anxiety Disorder Social Anxiety Disorder Night Terrors (Sleep Terror Disorder) Post-Traumatic Stress Disorder Sexual Trauma Anxiety-Related Disorders Sub-Total	2 7 3 1 2 6 3 1 4 2 31	24 60 14 11 12 60 25 1 25 12 244	3.58 (2.2–5.0) 8.94 (6.8–11.1) 2.09 (1.0–3.2) 1.64 (0.7–2.6) 1.79 (0.8–2.8) 8.94 (6.8–11.1) 3.73 (2.3–5.2) 0.15 (− 0.1–0.4) 3.73 (2.3–5.2) 1.79 (0.8–2.8) 36.31 (32.7–39.9)
Depressive Dis. (3 Major, 1 Mild/Moderate) Dysthymia Bipolar Disorder Mood Disorders Sub-Total	4 2 2 8	36 2 13 51	3.87 (2.4–5.3) 0.30 (− 0.1–0.7) 1.94 (0.9–3.0) 7.60 (5.6–9.6)
Internalizing disorders sub-total	39	295	43.96 (40.2–47.7)
ADHD (5 combined, 2 inattentive)	7	18	2.68 (1.5–3.9)
Enuresis Encopresis	7 1	18 1	2.68 (1.5–3.9) 0.15 (− 0.1–0.4)
Oppositional Defiant Disorder Conduct Disorder	7 1	9 1	1.34 (0.5–2.2) 0.15 (− 0.1–0.4)
Eating Disorders (1 Mild Binge-Eating Dis, 1 Anorexia Nervosa)	2	12	1.79 (0.8–2.8)
Tic Disorder	1	10	1.49 (0.6–2.4)
Substance Use Disorder (Poly-substance Dependence)	1	1	0.15 (− 0.1–0.4)
Externalizing disorders sub-total	27	70	10.43 (8.1–12.7)

*Estimated weighted prevalence not summed up to 100% because of considerable overlap in co-occurrence of multiple individual disorders in the same individual participant

### Socio-demographic correlations with mental disorders in study sample

The results of the multivariate logistic regression involving all significant factors are shown in Table 6. Including all significant variables resulted in residual deviance of 132.96 and AIC number 170.96. Removing the variable ‘internet access' (which was significant on chi-square analysis) from the model though resulted in only a slightly increased residual deviance of 133.42 and a decreased AIC of 169.42, indicating that the model was rendered more accurate by removing ‘internet access' from it. For all other variables though, removing them resulted in either an increased AIC number or an increased residual variance number, indicating a less accurate model than what was finally settled on.

**Table 6:** Binary Logistic Regression and Odds Ratio showing significant variables independently predictive of Mental Disorders

Variable	Estimated coefficient	P-value	Odds Ratio OR	95% Confidence Interval for OR
*Religious influence*				
Low Vs High	1.340	0.074	3.9	0.816–16.4
*Father’s education*				
None Vs Secondary	−17.489	0.995	0	0–1.27e50
Basic Vs Secondary	0.216	0.700	1.24	0.417–3.84
Tertiary Vs Secondary	0.309	0.721	1.36	0.186–6.57
*Mother’s education*				
None Vs Secondary	0.969	0.245	2.64	0.485–13.9
Basic Vs Secondary	0.409	0.544	1.51	0.42–6.30
Tertiary Vs Secondary	− 15.324	0.992	0	0–3.15e26
*Marital Status*				
Never married Vs married	0.977	0.466	2.66	0.1–29.7
Divorced Vs married	− 0.208	0.781	0.812	0.151–3.10
Widowed Vs Married	0.504	0.575	1.66	0.229–8.39
Active reading habit No Vs Yes	1.051	**0.059**	**2.86**	**1.01–9.3**
Wealth Index category 1st & 2nd quintiles (0–40) Vs 5th (80.1 –100)	2.599	0.140	13.5	0.333–605.00
3rd quintile (40.1–60) Vs 5th (80.1–100)	2.522	**0.040**	**12.5**	**1.39–274.**0
4th quintile (60.1–80) Vs 5th (80.1–100)	2.189	**0.039**	**8.93**	**1.66–167**
Repeated class No Vs Yes	0.802	0.377	2.23	0.301–11.8
Homework supervision < 11 years Infrequent Vs frequent	1.265	**0.023**	**3.54**	**1.16–10.5**

Bold values indicate the statistically significant or near significant at p ≤ 0.05

**Fig. 2:**
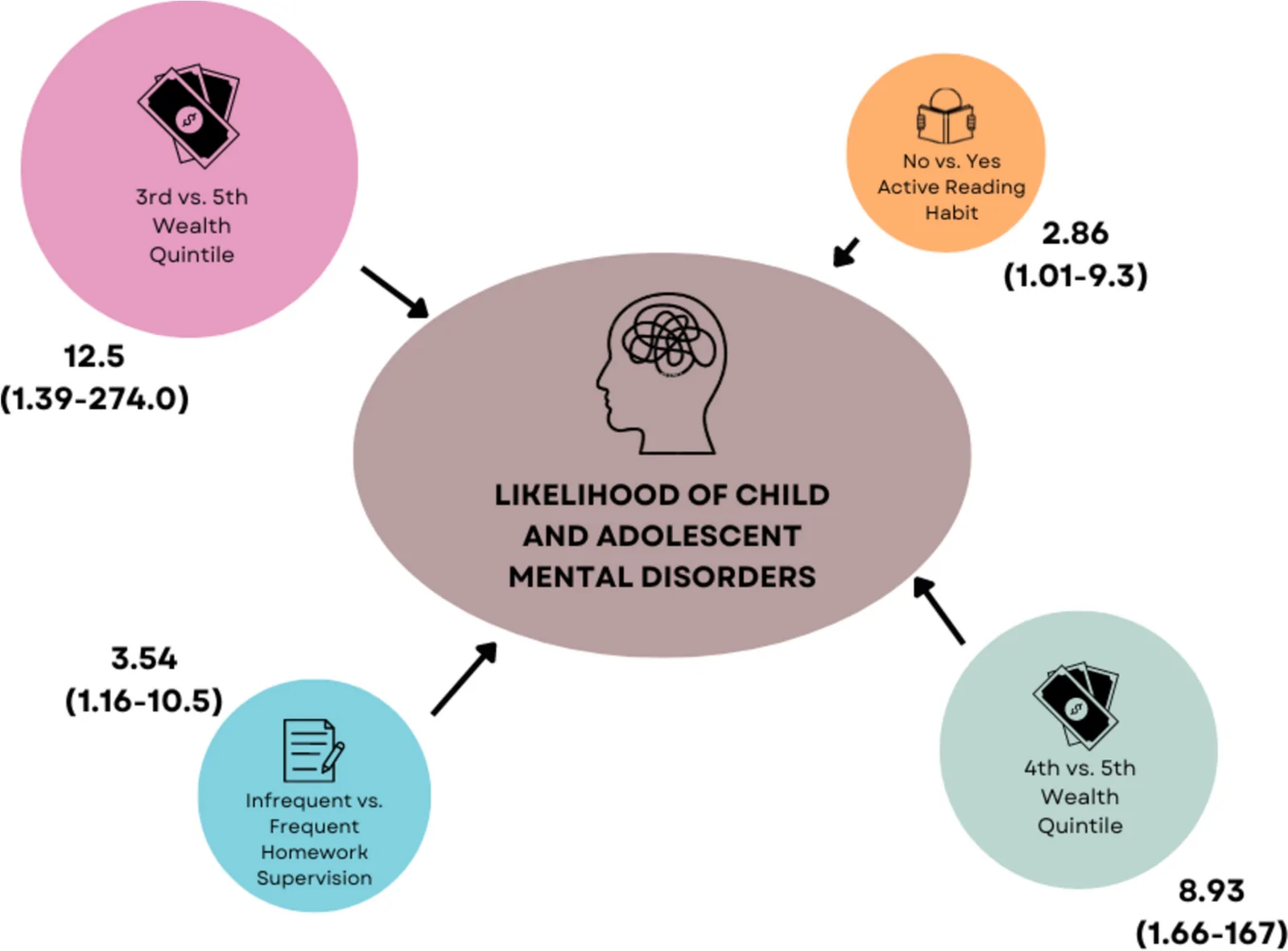
Factors independently predictive of presence of CAMH disorder and their Odds Ratios

Children belonging to the 3rd quintile of Wealth Index (IWI score 40.1 –60) were 12.5 times more likely than those in the 5th (highest) quintile to have mental disorders when controlling for other significant variables (*p* = 0.040; 95% CI 1.39- 274.0). Further, children belonging to the 4th quintile (IWI 60.1–80) were also 8.93 times more likely than those in the 5th quintile to have mental disorders (*p* = 0.039; 95% CI 1.66–167). Among children aged < 11 years in primary school, children whose caregivers reported infrequent supervision of homework by caregivers were 3.54 times more likely to have mental illness than those whose caregivers reported frequent supervision (*p* = 0.023; 95% CI 1.16–10.5). Also, children reporting having no active reading habit were 2.86 times more likely to have mental disorder than their counterparts who reported having an active reading habit (*p* = 0.059; 95% CI 1.01–10.5). These results are summarised in Fig. 2 below.

## Discussion

This study was a community-based cross-sectional epidemiological survey to assess the prevalence, pattern, and correlates of mental disorders among children and adolescents aged 6–17 years in an urban community in Kumasi, Ghana. To the knowledge of the researchers, this is the first community survey of child and adolescent mental disorders in Ghana.

### Prevalence and Pattern of Mental Disorders in Sample Population

Our weighted lifetime prevalence of 30.4% is significantly higher than the 10% of children and adolescent who experience a mental disorder as stated by the World Health Organization [63], although our weighted current prevalence of 18.6% is still within the 10–20% found in the earlier WHO Atlas Project [10]. The lifetime prevalence may have been higher though because this was an exclusively urban sample. The most recent literature from the past decade provides a mixed picture of the prevalence of CAMH disorders in rural versus urban youth, with studies showing higher prevalence in rural areas in certain disorders (e.g. substance misuse) while urban showed higher in other disorders (e.g. suicidal ideation) [64] and depression and anxiety [65]. Recent studies carried out in The Gambia in an urban area [66] and in a rural area in Eritrea [67] using a similar two-stage methodology obtained weighted current prevalence rates of 23.7% and 13.1%, respectively, again showing the rural urban disparities. A recent meta-analysis of community-based prevalence studies from sub-Saharan Africa reported a prevalence of 14.5% of mental disorders [27]. The weighted lifetime prevalence rate obtained in this study is understandably higher as this includes all mental disorders whether from the past or current (within the preceding 12 months), but is also in keeping with worldwide lifetime prevalence figures which range from 25–34% [43]). Overall, the results from this present study were comparable to community-based data reported from within Africa and around the world.

The literature corroborates our finding of internalizing disorders such as anxiety-related and mood disorders as having the highest prevalence, with an afore-mentioned meta-analysis also listing emotional and anxiety-related problems as the commonest disorders in the community [12, 27]. Although Anxiety disorders are the most common psychiatric disorders everywhere in the world, the high prevalence of anxiety-related disorders in Ghana was a bit surprising because they are not commonly reported to hospitals. Perhaps this is because most do not seek help early, probably because they are chronic and not life-threatening or overtly disruptive. In any case, this suggests there may be an unreported "silent epidemic" of these disorders in the community. The reasons for this lack of reporting could include a lack of recognition of anxiety disorders as mental health issues, as well as cost, accessibility, and other barriers to mental healthcare. In any case, this finding of a high prevalence of anxiety disorders in our exclusively urban sample is in keeping with findings from a meta-analysis from Australia where urban youth had higher rates of anxiety and depression than rural youth [65]. This finding has important implications for public policy and health communication in low-income settings, highlighting the need for further research to understand and address these barriers to care.

### Sociodemographic Correlates of DSM-5 Disorders Among Sample Population

Analysis of sociodemographic correlates with mental disorder in the sample population generated interesting results. Unlike in this study, other studies have reported significant associations with gender and age [35, 68], although overall this does not appear to be the case as noted in a meta-analysis of studies done in sub Saharan Africa which did not find significant differences in gender [27]. The positive connection between the self-reported influence of religion on one’s behaviour and low incidence of mental disorders on bivariate analysis was another interesting finding that has been little explored in sub-Saharan Africa, despite the region's high religiosity. Research from other parts of the world suggests that strong religious commitment is often associated with lower rates of mental health disorders [69–71], which makes this a potentially interesting line of enquiry for this region.

The finding that survival work of children under 15 years was associated with significantly more mental disorders aligns with a recent meta-analysis, showing a strong association between child labour and mental disorders in LMICs [72]. The meta-analysis also noted that older children engaged in child labour were more resilient to mental disorders, which is consistent with this study's findings. It is known that children exposed to survival work are more vulnerable to the stressors and hazards of the work environment that tends to precipitate or exacerbate any underlying mental health vulnerabilities, making this an important finding for local labour regulators.

Parental educational attainment was significant on bivariate analysis in this study but not on logistic regression, mirroring findings in the UK [24, 25], and suggesting the parental education influence may be mediated by a third confounder such as poverty/wealth. The finding of low maternal education’s association with increased mental illness in children has also been found in similar LMIC settings to that of the present study and in HICs as well [28, 73, 74], but the same does not necessarily hold true for paternal educational levels. This low parental educational attainment association to childhood mental illness is unsurprising though because it has been linked with low socioeconomic status, unfavourable attitude towards modern health care and health seeking behaviour, poor child nutrition and other mental health hazards [75, 76]. The link between parental marital status and childhood mental disorders has been well established [26]. Divorce, separation [24, 31, 77], being unmarried [24], and parental death [78] are associated with a higher incidence of childhood mental disorders, as was found in this present study. In a study from Ethiopia, a similar association was found, with children of unmarried, divorced, separated, or widowed parents having significantly higher rates of mental disorders [30]. This speaks to the role of a stable household environment in the mental wellbeing of the children who occupy them.

Extensive literature documents a link between reading and mental disorders [79–82], independent of confounding factors like self-esteem [83], confirming our findings about the effect of reporting an active reading habit. Children with poor reading habits are more likely to have mental disorders, especially externalizing disorders like ADHD [84], possibly related to existing challenges at school and home from the externalizing disorders [79, 84]. The significant association between limited Internet access and higher rates of mental illness is also noteworthy, possibly influenced by living standards.

Understandably, we found that repeating a class and inadequate homework supervision for primary school children were associated with higher rates of mental disorders, which was consistent with existing literature [28, 31, 85–88]. This association could be due to pre-existing cognitive difficulties leading to academic challenges, which, in turn, contribute to mental health issues and class repetition. Alternatively, pre-existing mental health problems could hinder academic performance and result in class repetition, creating a complex interplay often challenging to untangle in terms of causality.

Ultimately though, having an active reading habit, Wealth Index, and homework supervision frequency were the only factors independently predictive of mental disorders when considering all other factors. The independent association of Wealth Index with mental disorders is consistent with the known link between poverty and mental disorders in the literature [89, 90, 91]. In other studies, such poverty-related factors such as food insecurity [76, 92] independently raise proportions of children with internalizing disorders from 19 to 83% [92], as well as other socio-emotional problems [91], while increased family income decreased oppositional defiant and conduct disorder [93]. In a study from Norway [89], family economy was indirectly associated with both externalizing and internalising problems through parental emotional well-being and parenting practices, suggesting that parental emotional well-being and parenting practices are two potential mechanisms through which low socioeconomic status is associated with child mental health problems [29, 89], although we did not directly measure parental emotional well-being in our study. The Family Stress Model [94] provides a theoretical framework for understanding how economic hardship affects family dynamics and child development. According to this model, financial difficulties create stress within the family, leading to parental emotional distress and potentially less effective parenting practices, which in turn negatively impacts children's mental health. This supports the idea that improved material well-being independently relates to better health outcomes for children and adolescents, in line with other studies that report a similar independent association.

The independent association between mental disorders and an active reading habit aligns with existing literature [79–82]. However, the finding that a lack of caregiver supervision for primary school children was independently associated with an increase in mental disorders may serve as a proxy measure for a broader mediating factor like parental neglect. It suggests that the absence of supervision might not directly cause mental disorders but rather signals neglect, which could lead to associated mental health problems. The link between parental neglect and mental health issues is well-documented [5, 5, 6, 6, 92].

### The Import of our Findings for Population-Level Interventions for Mental Wellbeing

Regarding CAMH in Ghana, our findings highlight the gravity of economic and sociocultural factors as determinants of mental health and wellbeing. Lund et al. [95] developed a conceptual framework which grouped such social determinants into demographic, economic, neighbourhood, environmental, and sociocultural domains, which potentially could affect mental wellbeing at the population level. Within this framework, the economic domain refers to wealth-related factors influencing susceptibility to mental illness, and the sociocultural domain refers to how societal organisation and relationships impact mental health risk and protection. The domains are further demarcated into proximal and distal factors which influence mental well-being [95]. Under the economic domain, the predictive nature of the Wealth Index in this present study can be characterised as a proximal factor as it relates to income and assets which directly impact an individual, particularly of the ‘urban poor’ variety. In the sociocultural context, the predictiveness of an active reading habit and the level of homework supervision can also be described as proximal factors, as they are linked to education and, potentially, social support.

The link between socioeconomic status/poverty and child mental health is an important one that appears to run through several studies and is worth highlighting. Poverty-related factors such as food insecurity and exposure to work at an early age increase the risk of children developing mental health problems [27, 72, 92, 96]. Despite the findings by Canino et al. [26] in the USA to the contrary, this link agrees with findings from all over the world [89–91]. As discussed earlier, the Family Stress Model [94] enhances our understanding of the mechanisms by which socioeconomic factors influence child mental health, underscoring the multifaceted impact of poverty on family well-being and child outcomes.

This all provide potential pressure points at which future interventions or policy-level change can be planned for the improvement of mental health of children. Channelling these social determinants into accelerators may propel the achievement of the goal of good mental health under SDG3. This paper hints at the potential effect that such macro-level variables have on childhood mental health. For example, Rose-Clarke et al. [97] proposed interventions such as cash transfers and social protection systems in the economic realm and school-based and social support interventions in the sociocultural domain. In a setting similar to that of the present study, Kusi-Mensah et al. [39] found that the additive effects of cognitive stimulation, no poverty and low student–teacher ratio as accelerator synergies benefited SDG targets of internet access and good mental health. Cognitive stimulation and no poverty as accelerators corroborate our findings regarding the predictive effects of active reading habit and wealth.

Further, school mental health programmes which have been shown to have some positive effects on adolescents emotional and behavioural wellbeing [98] can also be considered for this urban community. Finally, the fact that overwhelming most participants lived in two-parent homes can be leveraged to improve the mental health of this community. The power of parent and family-focused interventions (e.g., psychoeducation, parent and family-skills training, behavioural, psychosocial, and trauma-focused cognitive behavioural therapy) to be beneficial to child and youth mental health and well-being, as well as parenting behaviours and family functioning has been shown by several studies as reported in this systematic review [99]. These can be promoted through culturally appropriate avenues by being channelled for example through churches and other community faith-based organisation, to ensure their acceptability and local buy-in. To enhance child and adolescent mental health in Ghana, policy suggestions should therefore encompass promoting greater reading habits and initiatives aimed at improving socioeconomic status and resourcing parent and family-focused interventions.

### Strengths and Limitations

The strengths of this study include the community-based study design, as well as the inclusion of 10% of those who screened negative in the initial screening, which allowed for a much more accurate estimation of the true prevalence in the general population of mental disorders. Also, the wide age range of 6–17 years gave a broader overview of the issues under study. This is an improvement over the only other survey of transnational families reported from Ghana which was a school-based survey, and only looked at a narrower age range of 11–21-year-olds. Finally, the sampling method was random, allowing for a fair degree of generalizability of the study findings. Limitations of this study included the fact that only one urban community was surveyed. Although this was a representative community typical of a Ghanaian urban population, it would have strengthened the generalizability of the findings if multiple communities covering both rural and urban dwellers had been used. Also, the final sample size of 672 while above the calculated sample size was relatively small for such a community-based epidemiological survey.

## Conclusion

This pioneering study represents the first comprehensive community-based research on CAMHD in Ghana. It has established lifetime and current prevalence rates, revealing patterns of DSM-5 childhood disorders that align with global and sub-Saharan Africa prevalence rates. Notably, it highlights the potential underreporting of CAMH disorders in the community, particularly anxiety-related disorders, which may be widespread in the sub-region given the scarcity of community-based studies in similar populations. Now that we have such a baseline study from the general population, we highly recommend further CAMH prevalence studies in various “at risk” children including those with neurodevelopment disorders, chronic medical conditions (e.g. Sickle Cell, HIV etc.) in order to compare with such general population data, to more robustly show the differences. Furthermore, this research strengthens the evidence regarding sociodemographic correlates of mental illness in African children and hints at previously undiscovered risk factors, warranting further investigation. It has significantly illuminated the profile of CAMH disorders in Ghana and the West Africa sub-region, emphasizing the necessity for well-designed epidemiological studies to inform mental healthcare decisions in Africa. These sociodemographic correlates may have vital policy implications regarding interventions that may accelerate the achievement of the goal of mental health as part of SDG3. Additionally, it underscores the importance of conducting community needs assessments to enhance knowledge, access, and utilization of mental health services in LMIC settings. Finally, it underscores the need for deeper exploration into the lived experiences of African children to uncover the factors contributing to their apparent resilience.

## Summary

This study has examined the prevalence and correlates of mental disorders among children and adolescents in Kumasi, Ghana, through a community-based cross-sectional survey. It is the first such community-based prevalence of childhood mental disorders (to the best of authors’ knowledge) from Ghana. In this study, a sample of 672 urban participants aged 6–17 years were surveyed. Mental disorders were screened using Rutter’s A2 Scale for Parent Assessment of Child Behaviour, with diagnoses confirmed by the Kiddie-Schedule for Affective Disorders and Schizophrenia. The Double Sampling method was used for weighted prevalence estimates, and correlates analysed using chi-square and logistic regression. Lifetime weighted prevalence of CAMH disorders was 30.4% (95% CI: 26.9–33.9), which was made up mostly of anxiety-related disorders followed by mood disorders. Also, current weighted prevalence was 18.6% (95% CI: 15.7–21.5). Notably, lacking cognitive stimulation such as an active reading habit was associated with nearly three times the odds of mental illness. Also, compared to those in the highest wealth quintile, children in the 3rd and 4th wealth quintiles had significantly higher odds of mental disorder (12- and 9-times increased odds, respectively), indicating the predictive association of relative poverty for mental disorders among children. Similarly, did lack of caregiver homework supervision among children under 11 years was also independently predictive of childhood mental disorders. This study highlights the potential underreporting of CAMH disorders in the community, particularly anxiety-related disorders in Ghana. It also highlights the link between poverty-related factors and mental health and suggesting potential policy interventions to inform policy for attaining SDG 3. Finally, it’s a useful paper in that it presents data from a neglected part of the world that has very few such prevalence data published, but that could have important perspectives to contribute to the world’s literature.

## Data Availability

The datasets generated during and/or analysed during the current study can be made available on request of the PI.
